# Therapeutic Vasopressor Use for Postspinal Hypotension in Low-Risk Elective Cesarean Deliveries: A Systematic Review, Network Meta-Analysis, and Trial Sequential Analysis

**DOI:** 10.7759/cureus.89934

**Published:** 2025-08-12

**Authors:** Arya Babul, Sohi Ashraf, Leanne Free, Jyoti Desai, Momina Hussain, Najib Babul

**Affiliations:** 1 Biomedical Sciences, West Career &amp; Technical Academy, Las Vegas, USA; 2 Biomedical Sciences, Society for Awareness of Neglected Diseases (SAND), Las Vegas, USA; 3 Intensive Care, MountainView Hospital, Las Vegas, USA; 4 Gynecologic Surgery and Obstetrics, Kirk Kerkorian School of Medicine at University of Nevada, Las Vegas (UNLV), Las Vegas, USA; 5 Genomics, Chinese Academy of Tropical Agricultural Sciences, Sanya, CHN; 6 Drug Development, Quadra Therapeutics, Las Vegas, USA

**Keywords:** low-risk elective cesarean delivery, network meta-analysis, norepinephrine, spinal anesthesia, vasopressors

## Abstract

Spinal anesthesia-induced hypotension (SAIH) is a common complication of cesarean delivery (CD), potentially leading to maternal discomfort and fetal compromise. Vasopressors such as norepinephrine (NE), phenylephrine (PE), and ephedrine (EP) are frequently used for treatment, yet their comparative efficacy and safety remain uncertain. This study aimed to assess and compare the effectiveness and tolerability of NE, PE, and EP for managing postspinal hypotension (PSH) in low-risk elective CD.

Systematic searches were conducted in PubMed, Embase, Cochrane CENTRAL, ScienceDirect, and ClinicalTrials.gov through June 2025. The protocol was registered in the International Prospective Register of Systematic Reviews (PROSPERO) (CRD420251074831). We included randomized controlled trials (RCTs) involving parturients undergoing low-risk elective cesarean section who received NE, PE, or EP for the management of PSH.

A systematic review, network meta-analysis (NMA), and trial sequential analysis (TSA) were performed. The primary outcome was the successful correction of PSH. Secondary outcomes included maternal bradycardia, nausea, vomiting, neonatal Apgar scores, and umbilical artery pH. Risk of bias was assessed using the Cochrane RoB 2 tool, and the certainty of evidence was graded with the GRADE (Grading of Recommendations Assessment, Development and Evaluation) methodology.

A total of 16 RCTs encompassing 2,102 parturients were included. NE demonstrated superior efficacy in reversing PSH (odds ratio (OR): 0.23; 95% confidence interval (CI): 0.09-0.58) and was associated with fewer adverse maternal events, including bradycardia (OR: 0.28) and nausea/vomiting (OR: 0.36), compared to PE and EP. Neonatal outcomes were generally comparable across groups, though NE showed a favorable trend in reducing the risk of neonatal acidosis (umbilical artery pH OR: 1.25; 95% CI: 1.06-1.54). Surface under the cumulative ranking curve (SUCRA) rankings and TSA supported the robustness of these findings. NE appears to be the most effective and best-tolerated vasopressor for treating SAIH during elective CD, without compromising neonatal safety. These results support the preferential use of NE over PE and EP in this clinical setting.

## Introduction and background

Spinal anesthesia (SA) remains the preferred anesthetic technique for elective cesarean delivery (CD) due to its safety, rapid onset and recovery, and acceptable maternal and neonatal outcomes [1,2]. However, SA is commonly associated with postspinal hypotension (PSH), with an incidence as high as 60% to 80% in the absence of prevention [3]. PSH results primarily from sympathetic blockade, leading to vasodilation, decreased systemic vascular resistance, and venous pooling [4]. If not corrected, maternal PSH can result in detrimental effects such as nausea (N), vomiting (V), dizziness, reduced uteroplacental perfusion, and compromised fetal oxygenation-clinically demonstrated by fetal acidosis (FA) or low Apgar scores [5,6].

Vasopressors (VPs) are a cornerstone for the management of PSH. Phenylephrine (PE), norepinephrine (NE), and ephedrine (EP) are commonly used agents [7,8]. Although PE has been the agent of longstanding choice owing to its potent α-adrenergic vasoconstrictor activity, it is accompanied by reflex bradycardia and reduced cardiac output. NE, a mixed α- and mild β-agonist, is a potentially superior agent with the theoretical advantage of maintaining vascular tone and cardiac output. Once favored, EP is disfavored due to an increased risk of FA associations [9-11]. While numerous randomized controlled trials (RCTs) have compared these medications, there is no consensus on the preferred VP for the management of PSH in low-risk elective CD [12-14].

Systematic reviews (SRs) conducted in the recent past have compared prophylactic VP strategies or contrasted a solitary agent in isolation, but not the comparative effectiveness of several VPs under a common evidence framework. Most SRs have been limited to pairwise contrasts and lack adequate statistical robustness to quantify the sufficiency of available evidence, due to sparse data, multiple hypothesis testing, and potential random error [15,16]. To the best of our knowledge, few trials to date have applied trial sequential analysis (TSA) to determine the conclusiveness of results in this clinical context, and no trial has utilized both network meta-analysis (NMA) and TSA in the same overarching framework for the therapeutic (as opposed to prophylactic) use of VPs among low-risk obstetric patients [17,18].

Moreover, given the evolving nature of obstetric anesthesia and recent shifts in VP usage trends, it is essential to continuously re-evaluate clinical evidence to inform best practices. Greater utilization of NE in clinical practice is guided by developing but nonetheless incomplete evidence [19]. Some RCTs have provided evidence of favorable hemodynamic profiles and enhanced maternal cardiac output with NE compared to PE. Outcomes, however, have been mixed, and variations in study populations, routes of administration (bolus vs. infusion), adjunctive interventions (fluid co-loading), and trial endpoints have contributed to considerable heterogeneity across studies [14].

To address this divergence, we conducted a SR, NMA, and TSA of RCTs to compare the efficacy of PE, NE, and EP in the treatment of PSH during low-risk elective CD. Our primary objective was to compare the efficacy of these VPs in correcting PSH. Secondary outcomes included maternal safety (N and bradycardia) and fetal/neonatal well-being (umbilical artery pH (UA pH), Apgar scores). Using the surface under the cumulative ranking curve (SUCRA), we determined the relative efficacy and safety of each agent. TSA was employed to assess the robustness and conclusiveness of the pooled effect estimators.

## Review

Methods

This systematic review and network meta-analysis (SRMA) was conducted in line with the Preferred Reporting Items for Systematic reviews and Meta-Analyses (PRISMA)-NMA and PRISMA 2020 guidelines [20]. The study protocol was registered in the International Prospective Register of Systematic Reviews (PROSPERO) under number CRD420251074831. The PRISMA checklist is provided in Appendix A.

Literature search strategy

A systematic search was performed using PubMed, Embase, the Cochrane Central Register of Controlled Trials (CENTRAL), Scopus, and Web of Science. The search strategy employed both Medical Subject Headings (MeSH) and free-text terms related to CD, SA, and VP agents. An example of a PubMed search string included the following: "(vasopressor* OR phenylephrine OR norepinephrine OR ephedrine) AND (PSH OR ""low blood pressure"") AND (""cesarean section"" OR ""caesarean section"" OR ""cesarean delivery"") AND (""spinal anesthesia"" OR ""subarachnoid block"") AND (treatment OR therapeutic)". The full search strategy for at least one of the databases is documented in Appendix B. We selected PubMed, Embase, Cochrane CENTRAL, and Scopus for our systematic search strategy as they collectively offer comprehensive and overlapping coverage of peer-reviewed clinical research relevant to obstetric anesthesia. These databases are recognized for their indexing of RCTs and high-impact journals. Although additional databases such as Web of Science and gray literature sources were considered, our scoping search revealed high redundancy and minimal added yield. Moreover, in alignment with our predefined protocol, we excluded gray literature due to concerns over a lack of peer review and quality appraisal limitations, which could compromise the methodological rigor and reproducibility of our findings.

Eligibility criteria

The included studies were RCTs comparing the therapeutic use of VPs for the management of PSH during elective CD in low-risk women. Trials were eligible if they enrolled women who were of low obstetric risk and had spinal or combined spinal-epidural anesthesia. Trials comparing more than one VP, PE, NE, and EP-VPs and comparisons of VPs with placebo, usual care, or other VPs were included. Trials were required to report at least one primary or secondary maternal or neonatal outcome. Non-randomized, animal studies, case reports, reviews, and conference abstracts were excluded. No date restrictions were applied, and studies from database inception to May 2025 were included. This review excluded trials that administered VPs prophylactically (i.e., prior to the onset of hypotension) and studies enrolling high-risk obstetric patients (e.g., pre-eclampsia and multiple gestation). Although our search strategy did not impose language restrictions, the vast majority of eligible studies were published in English. Non-English-language articles were screened and assessed for eligibility; however, they were either duplicates of English publications or failed to meet our inclusion criteria (e.g., insufficient data and non-randomized design). Therefore, their exclusion had minimal to no impact on the comprehensiveness of the evidence base. We have clarified this in the Methods section to maintain transparency.

Study selection

**Figure 1:**
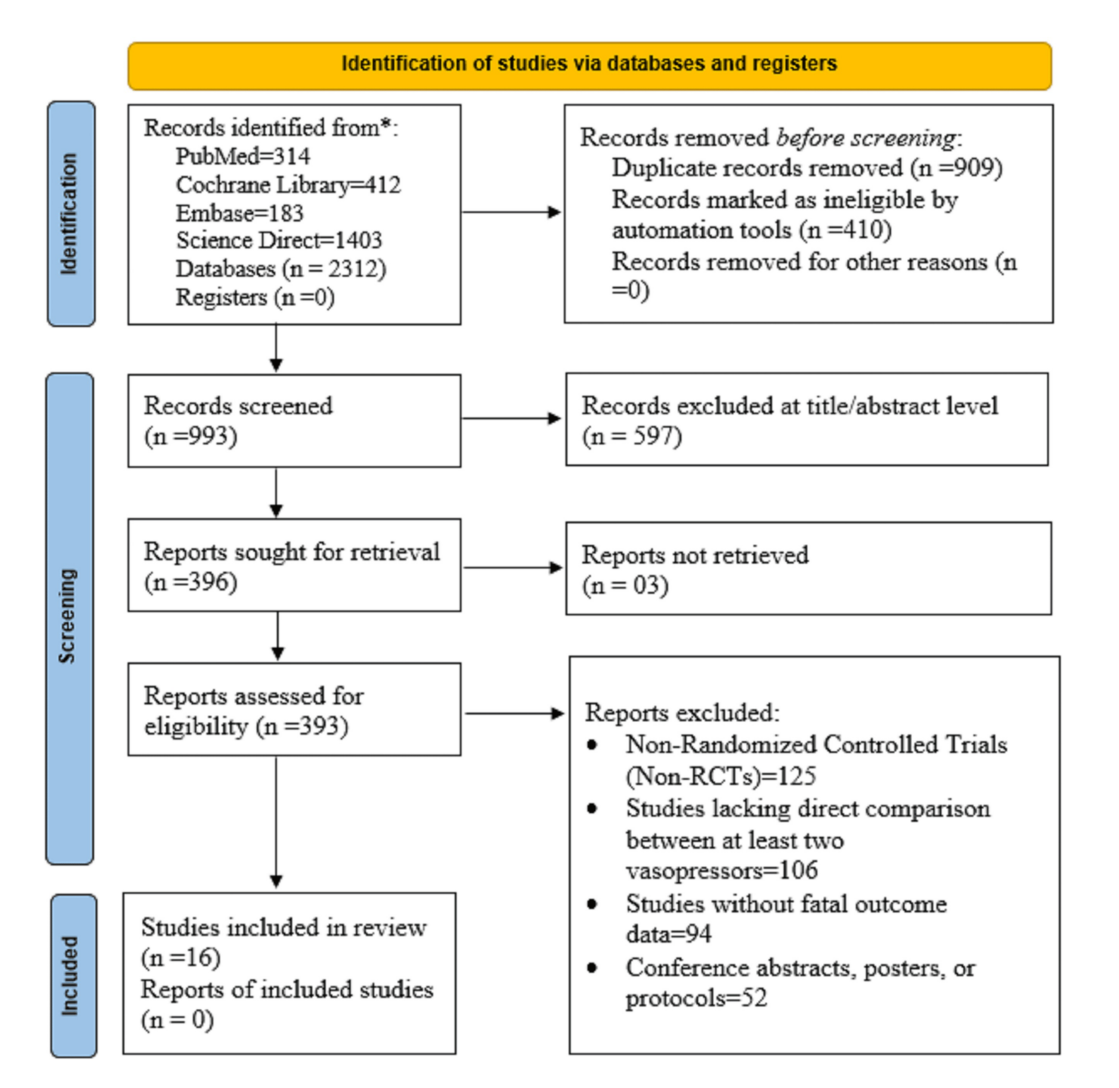
PRISMA flow diagram PRISMA: Preferred Reporting Items for Systematic reviews and Meta-Analyses

Two independent reviewers (AB and NB) screened the titles and abstracts for relevance. The full text of potentially relevant studies was subsequently assessed against the inclusion and exclusion criteria. Disagreements were resolved through discussion or by a third reviewer (MH). A PRISMA flow diagram was used to document the selection process and reasons for the exclusion of studies (Figure 1).

Data extraction and quality assessment

Data were independently extracted by two reviewers using a pilot-tested standard extraction form [21]. The following information was collected: first author, year of publication, country, sample size, description of the intervention and comparator (type of VP, route of administration, and dosing), maternal outcomes (frequency of resolution of PSH, hemodynamic stability, bradycardia, N, and V), and neonatal outcomes (one- and five-minute Apgar scores, UA pH, frequency of acidosis, or low Apgar scores). Additional information on adjunct therapies such as fluid co-loading was collected. Conflicts in data extraction were resolved by consensus.

Outcomes of interest

The primary outcome of interest was the resolution of PSH, defined as the resolution of hypotension following SA, in low-risk elective cesarean section. Secondary maternal outcomes were the rate of bradycardia, N, and V. Neonatal outcomes included the Apgar score at one and five minutes, UA pH, and incidence of neonatal acidosis (defined as UA pH < 7.20). Where feasible, composite neonatal health outcomes from trials publishing or requiring rescue VP administration were also tabulated. All results were grouped and examined according to predefined definitions reported in the original trials or accepted clinical thresholds.

Statistical analysis

Risk of Bias Assessment

Risk of bias (RoB) in methods was assessed using the Cochrane Risk of Bias tool 2.0 (RoB 2.0) [22]. All trials were assessed across five domains: process of randomization, deviations from planned interventions, missing data on the outcome, measurement of the outcome, and selection of the reported result. Each domain was rated as having a low risk, some concerns, or a high RoB. The overall RoB for each study was determined. The overall certainty of the evidence was assessed using the GRADE (Grading of Recommendations Assessment, Development and Evaluation) system [23]. All authors agreed on the final grades, which were assigned as follows: high (⊕⊕⊕⊕), moderate (⊕⊕⊕⊖), low (⊕⊕⊖⊖), or extremely low (⊕⊖⊖⊖).

Data Synthesis

Pairwise meta-analyses were conducted using a DerSimonian-Laird random effects model to account for between-study heterogeneity. All odds ratios (ORs) were calculated such that an OR < 1 favors the intervention (NE), with the outcome defined as persistent hypotension. A network frequentist meta-analysis was performed using the netmeta R package (R Foundation for Statistical Computing, Vienna, Austria), which allows simultaneous comparison of several VPs in a network of evidence. Relative treatment effects were expressed as ORs with 95% confidence intervals (CIs). SUCRA probabilities of efficacy and safety outcomes were computed to rank interventions [24].

Trial Sequential Analysis

To calculate the risk of random errors and determine whether cumulative proof was sufficient, TSA used TSA software (version 0.9, Copenhagen Trial Unit, Denmark) [25]. The parameters used were a two-sided alpha level of 0.05 and a beta of 0.20 with the employment of a random-effects model. Required information size (RIS) was calculated, adjusting for heterogeneity using the diversity (D²) measure. TSA was also performed on the primary outcome in studies judged to have a low RoB.

Results

Study Selection and Characteristics

A total of 2,312 records were identified in the early searches of four primary databases: PubMed (n = 314), Cochrane Library (n = 412), Embase (n = 183), and Science Direct (n = 1,403). Prior to screening, 909 duplicate records were removed, and 410 records were removed using automated software, thus leaving 993 exclusive records for title and abstract screening. During screening, 597 records were excluded because they were irrelevant or did not meet the inclusion criteria. Subsequently, 396 reports were asked for full-text retrieval; however, three were unavailable. Of the 393 full-text articles screened for eligibility, 377 were excluded for the following reasons: 125 were non-RCTs, 106 lacked direct comparisons of at least two VPs, 94 did not provide fetal outcome results, and 52 were conference abstracts, posters, or protocols that were not completed (Figure 1). In total, 16 RCTs involving 2,102 low-risk parturients undergoing CD under SA were included (Table 1). Studies conducted between 2005 and 2025 across Iran, Turkey, Korea, India, Brazil, Egypt, the UK, and Hong Kong provided a diverse yet comparable dataset. All participants were American Society of Anesthesiologists Physical Status Classification I-II (ASA I-II), with singleton term pregnancies undergoing planned CD. Three VPs, PE, EP, and NE, were administered either as an intravenous (IV) bolus (PE 50-100 µg, EP 5-10 mg, and NE 4-6 µg) or continuous infusion (PE 25-100 µg/min, NE 2-5 µg/min). Overall, 397 patients received VP as the first treatment for the alleviation of SA-induced PSH. The remaining subjects were placed in comparator groups with the administration of other VPs or standard protocols. Adjunctive procedures such as crystalloid preload/co-load and intrathecal bupivacaine with opioid additives (fentanyl and sufentanil) were administered equally. Maternal effects were primarily bradycardia, N, and V, and neonatal outcomes were Apgar scores and UA pH. PE was frequently associated with a higher incidence of bradycardia, whereas NE had an improved profile with lower incidences of bradycardia and N. In all studies, five-minute Apgar scores were ≥7 and UA pH levels were generally >7.20, demonstrating no clinically relevant neonatal acidosis. These studies constitute a consistent and high-quality foundation for conducting NMA and TSA of VP efficacy and safety in low-risk CD under SA.

**Table 1:** Characteristics of included RCTs evaluating vasopressor use in low-risk elective CD under SA CD: cesarean delivery; SA: spinal anesthesia; IV: intravenous; ASA I-II: American Society of Anesthesiologists Physical Status Classification I–II; SBP: systolic blood pressure; EP: ephedrine; PE: phenylephrine; NE: norepinephrine; UA pH: umbilical artery pH; RCTs: randomized controlled trials; N: nausea; V: vomiting

Author	Year	Country	N	Population	Intervention	Comparator	VV	Mode	Adjunct therapy	Bradycardia	N/V	Apgar (1 & 5 min)	UA pH/neonatal acidosis
Amiri et al. [26]	2013	Iran	100	Term singleton, elective CD, SA	PE 100 µg IV bolus	EP 10 µg IV bolus	PE vs. EP	Bolus	RL preload, oxygen via mask	PE: 18%, EP: 12%	N: 4%, V: 4% in EP group	1 min: mostly ≥7; 5 min: all ≥9	pH similar; PE had higher BE
Biricik et al. [27]	2020	Turkey	160	ASA I-II elective CD, SA	EP 5 µg/mL IV infusion	PE 100 µg/mL, NE 5 µg/mL, saline (placebo)	EP vs. PE, NE	Infusion	RL co-load, rescue EP for SBP < 80% baseline	NE: 12.5%, E: 7.5%, PE: 15%, saline: 12.5%	N: 12.5%-20%, V: 7.5%-15% (across groups)	1 min: E: 9, others: 8; 5 min: E: 10, others: mostly 9	NE: 7.34, EP: 7.32, PE: 7.31, saline: 7.3; no severe acidosis
Cho et al. [28]	2020	Korea	56	Healthy parturients, elective CD, SA	NE infusion (0.05 µg/min)	PE infusion (0.5 µg/min)	NE vs. PE	Infusion	Crystalloid co-loading, spinal bupivacaine with fentanyl	NE: 0%, PE: 22.5%	N/V NE: 5%, PE: 15%	1 min: ≥8; 5 min: all ≥9 in both groups	UA pH: NE: 7.32 ± 0.04, PE: 7.31 ± 0.05; no significant acidosis
Das et al. [29]	2025	India	102	ASA I-II, term elective CD	NE (5 µg IV bolus)	PE (100 µg IV bolus)	NE vs. PE	Bolus	Co-load with Ringer lactate, spinal bupivacaine with fentanyl	NE: 4%, PE: 16%	NE: 6%, PE: 12%	1 min: ≥8; 5 min: all ≥9 in both groups	UA pH: NE: 7.30 ± 0.03, PE: 7.28 ± 0.04; no neonatal acidosis reported
de Queiroz et al. [30]	2023	Brazil	76	Elective CD, SA	NE (5 µg/mL) IV infusion	PE (100 µg/mL) IV infusion	NE vs. PE	Infusion	Crystalloid co-loading; SA with bupivacaine/fentanyl	NE: 0%, PE: 29.2%	NE: 10%, PE: 15%	1 min: all ≥8; 5 min: all ≥9	UA pH: NE: 7.31, PE: 7.29; no neonatal acidosis
Gunda et al. [31]	2010	India	100	Healthy, elective CD, SA	EP 10 µg IV bolus	PE 100 µg IV bolus	EP vs. PE	Bolus	Ringer lactate preload; SA with hyperbaric bupivacaine	PE: 13.3%, EP: 0%	PE: 3.3%, EP: 26.7%	1 min: all ≥7; 5 min: all ≥9	PE: 7.31, EP: 7.28; no acidosis reported
Hassabelnaby et al. [32]	2020	Egypt	110	Healthy, term, elective CD, SA	NE (4 µg) IV bolus	PE (100 µg) IV bolus	NE vs. PE	Bolus	Ringer lactate co-load; SA with bupivacaine/fentanyl	NE: 5%, PE: 18%	NE: 6%, PE: 13%	1 min: ≥8; 5 min: ≥9 in both groups	UA pH: NE: 7.32 ± 0.02, PE: 7.30 ± 0.03; no acidosis
Kansal et al. [33]	2005	India	60	ASA I-II, elective CD	EP 10 µg IV bolus	PE 100 µg IV bolus	EP vs. PE	Bolus	Ringer lactate preload; spinal bupivacaine anesthesia	PE: 10%, EP: 0%	PE: 6.7%, EP: 26.7%	1 min: all ≥7; 5 min: all ≥9	PE: 7.32 ± 0.04, EP: 7.29 ± 0.03; no neonatal acidosis
Macfarlane et al. [34]	2009	United Kingdom	70	ASA I-II, term, elective CD, SA	PE 100 µg/mL IV infusion	EP 5 µg/mL IV infusion	PE vs. EP	Infusion	Co-load of Hartmann’s solution, SA with bupivacaine and fentanyl	PE: 26%, E: 16%	PE: 26%, E: 16%	1 min: median ≥8; 5 min: median 10 both groups	PE: 7.29 ± 0.04, E: 7.29 ± 0.04; no acidosis
Mohta et al. [35]	2015	India	120	ASA I-II, elective CD, SA	NE 5 µg IV bolus	PE 100 µg IV bolus	NE vs. PE	Bolus	Ringer lactate co-load; SA with bupivacaine/fentanyl	NE: 3.3%, PE: 11.7%	NE: 5%, PE: 15%	1 min: ≥8; 5 min: ≥9	UA pH: NE: 7.32, PE: 7.29; no acidosis reported
Mohta et al. [36]	2019	India	100	Term, elective CD, SA	PE 100 µg IV bolus	EP 10 µg IV bolus	PE vs. EP	Bolus	Crystalloid preload; SA with bupivacaine/fentanyl	PE: 13.3%, EP: 3.3%	PE: 6.7%, EP: 20%	Both groups: 1 min ≥8; 5 min ≥9	PE: 7.32 ± 0.02, EP: 7.30 ± 0.03
Mohta et al. [37]	2019	India	90	ASA I-II, term, elective CD	NE 4 µg IV bolus	PE 50 µg IV bolus	NE vs. PE	Bolus	Crystalloid preload; spinal bupivacaine + fentanyl	NE: 3.3%, PE: 13.3%	NE: 6.7%, PE: 13.3%	Similar and acceptable in both groups	Comparable pH; no acidosis reported
Ngan Kee [38]	2017	Hong Kong	180	ASA I-II, CD, SA	NE infusion (5 µg/mL)	PE infusion (100 µg/mL)	NE vs. PE	Infusion	Crystalloid co-load, bupivacaine/fentanyl SA	NE: 0%, PE: 17%	NE: 6%, PE: 17%	Median 1 min: 9; 5 min: 10 (both)	NE: 7.31 ± 0.05, PE: 7.30 ± 0.05; no acidosis
Ngan Kee et al. [39]	2020	Hong Kong	668	Healthy, term, elective CD, SA	NE 5 µg/mL infusion	PE 100 µg/mL infusion	NE vs. PE	Infusion	Co-load of lactated Ringer, spinal bupivacaine + fentanyl	NE: 0%, PE: 22%	NE: 7%, PE: 15%	1 min: 9; 5 min: 10 in both groups	NE: 7.30 ± 0.04, PE: 7.28 ± 0.05; no acidosis
Prakash et al. [40]	2010	India	60	ASA I, term, elective CD, SA	EP 6 µg IV bolus	PE 100 µg IV bolus	EP vs. PE	Bolus	Preload with RL, oxygen supplementation, bupivacaine spinal block	EP: 0%, PE: 16.7%	EP: 13%/3.3%, PE: 0%/0%	Both groups: ≥8 at 1 min, ≥9 at 5 min	EP: 7.29 ± 0.04, PE: 7.32 ± 0.04; lower BE in EP group
Puthenveettil et al. [41]	2019	India	50	ASA I-II, term, elective CD	NE 4 µg IV bolus	PE 50 µg IV bolus	NE vs. PE	Bolus	Ringer lactate preload; bupivacaine + fentanyl SA	NE: 4%, PE: 20%	Comparable between groups (not significant)	Similar in both groups	Comparable UA pH; no neonatal acidosis reported

RoB Assessment

**Figure 2:**
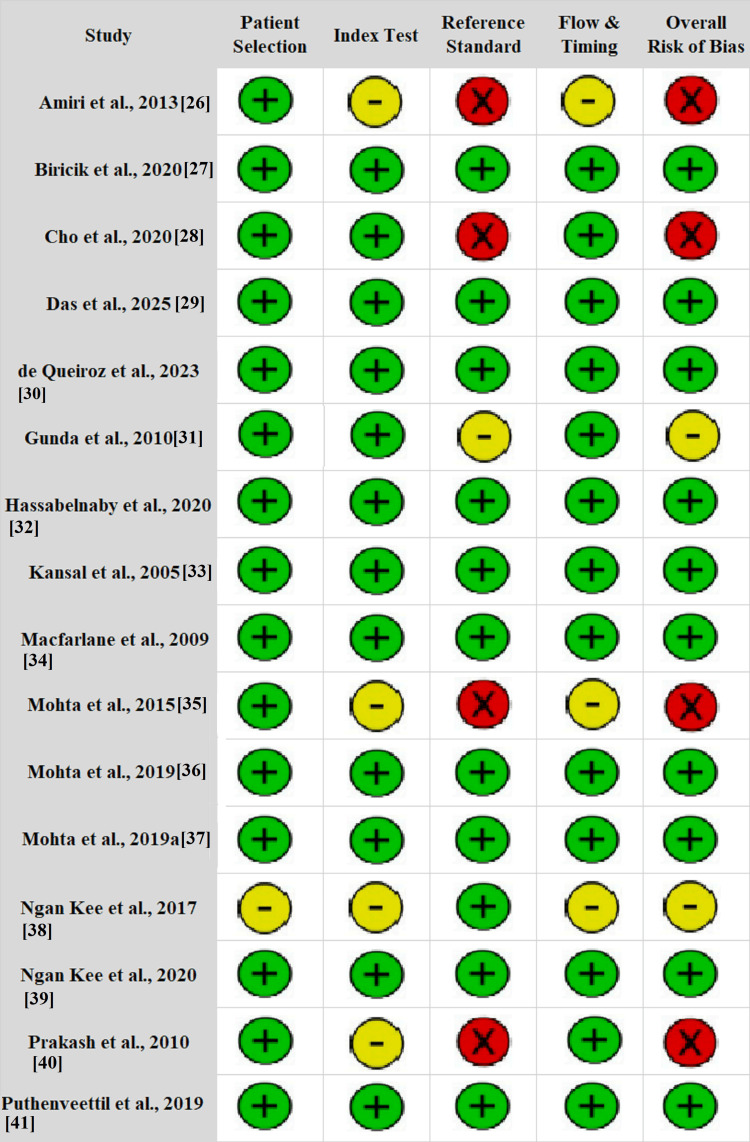
Risk of bias assessment of included studies using the QUADAS-2 tool Risk of bias was evaluated across four domains: patient selection, index test, reference standard, and flow & timing. An overall risk of bias judgment is also presented for each study Green circle with “+”: low risk of bias; yellow circle with “–”: unclear risk of bias; red circle with “×”: high risk of bias

RoB assessment across the 16 included RCTs indicated generally high methodological quality. In total, 10 studies [27,29,30,32-34,36,37,41,42] were rated as having low overall RoB, reflecting consistency and reliability in study design and execution. Two studies demonstrated some concerns, primarily due to unclear procedures related to the index test or issues in flow and timing [31,38]. Meanwhile, four studies [26,28,35,40] were rated as having a high RoB, mainly due to the lack of reference standard and lack of flow and timing reporting [26,35]. Overall, flow and timing were appropriate, with some studies reporting unclear time frames between intervention and outcome evaluation, which raised suspicions regarding the completeness or timing consistency of data (Figure 2). Overall, the evidence base is supported by the prevalence of low-risk studies, which justifies the findings of the NMA and TSA.

Quality of Evidence

The GRADE analysis revealed high certainty of evidence for the primary outcome of hypotension resolution (OR: 0.23; 95% CI: 0.09-0.58), no downgrades. Evidence on the incidence of bradycardia was downgraded from moderate due to heterogeneity between studies. Similarly, the outcome of N/V incidence was supported by moderate-certainty evidence, affected by imprecision and potential small-study effects. On the contrary, the UA pH/neonatal acidosis outcome was assigned low certainty, on the basis of concerns in terms of RoB and heterogeneity of outcome definitions and measurement strategies (Table 2).

**Table 2:** Summary of findings and quality of evidence (GRADE) for primary and secondary outcomes GRADE: Grading of Recommendations Assessment, Development and Evaluation; CI: confidence interval; UA pH: umbilical artery pH

Outcome	No. of studies	RR (95% CI)	Certainty (GRADE)	Downgrade reason
Hypotension resolution	16	RR 0.23 (0.09–0.58)	High ⨁⨁⨁⨁	None
Bradycardia incidence	16	RR 0.31 (0.17–0.57)	Moderate ⨁⨁⨁◯	Inconsistency due to heterogeneity
Nausea/vomiting incidence	16	RR 0.47 (0.25–0.89)	Moderate ⨁⨁⨁◯	Imprecision and possible small-study effects
UA pH/neonatal acidosis	16	RR 1.25 (0.85–1.83)	Low ⨁⨁◯◯	Risk of bias and imprecision

Meta-analysis

Maternal Outcomes

**Figure 3:**
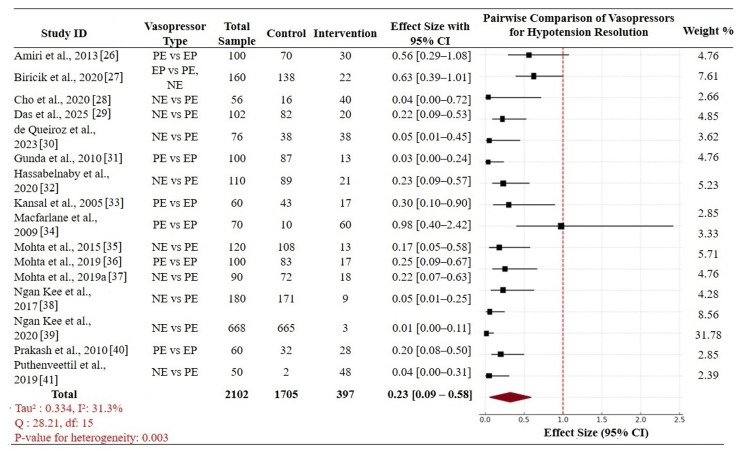
Forest plot: pairwise comparison of vasopressors for PSH resolution PSH: postspinal hypotension; PE: phenylephrine; NE: norepinephrine; EP: ephedrine; CI: confidence interval

Postspinal hypotension: A pairwise meta-analysis of the primary outcome assessed PSH persistence among 16 RCTs with comparator direct comparisons of several VPs. A total of 2,102 parturients were recruited, 397 for the VP under investigation and 1,705 for a comparator VP. Comparators were principally between NE vs. PE, PE vs. EP, and NE vs. EP. The overall pooled effect size across the persistence of PSH highly favored the intervention groups with an OR of 0.23 (95% CI: 0.09-0.58), showing a very large reduction in the odds of persistent PSH in patients who received the intervention VPs. Heterogeneity was moderate (Tau² = 0.334; I² = 31.3%), and Cochran's Q-statistic (Q = 28.21, df = 15) detected statistically significant heterogeneity (p = 0.003). Of special interest, Ngan Kee et al. [39] and Puthenveettil et al. [41] showed extreme strength in the support for NE over PE, with ORs of 0.01 (95% CI: 0.00-0.11) and 0.04 (95% CI: 0.00-0.31), respectively, capturing 31.78% and 2.39% of the total weight [41,42]. In contrast, previous studies such as Macfarlane et al. [34] yielded non-significant results (OR = 0.98 (95% CI: 0.40-2.42)), contributing minimally to the pooled estimate. These results underlie the higher efficacy of NE in avoiding recurrent PSH against PE and EP, supporting its value as a first-line VP in low-risk cesarean section under SA (Figure 3) [34].

**Figure 4:**
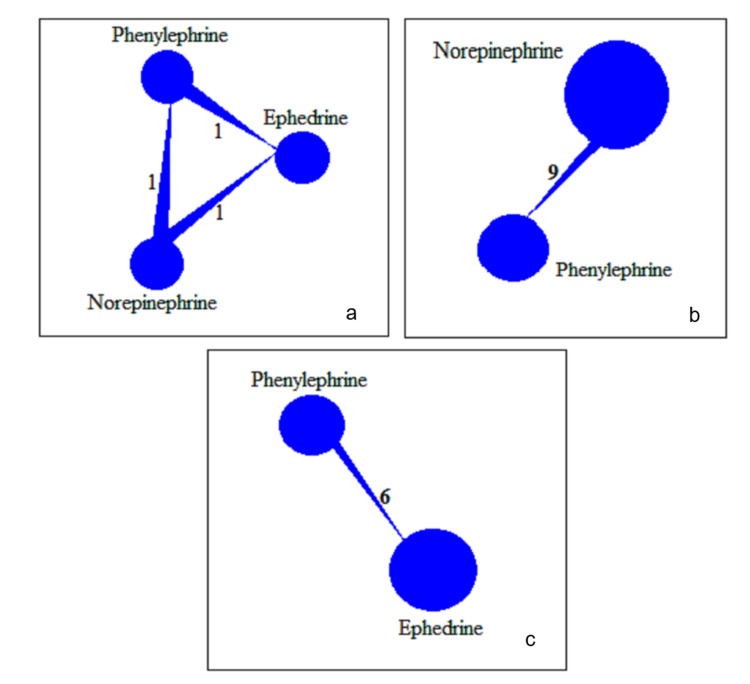
Network plots of treatment comparisons among norepinephrine, phenylephrine, and ephedrine for maternal hypotension management (a) Overall network geometry depicting all available direct comparisons across studies. (b) Network plot for studies using norepinephrine vs. phenylephrine. (c) Network plot for studies using phenylephrine vs. ephedrine The size of each node is proportional to the total number of participants receiving that intervention across included studies. The thickness of the connecting lines reflects the number of direct comparisons between the treatments, with the numbers on each edge indicating the total number of studies for that comparison

NMA of PSH: The geometry of the studies graphically illustrates the shape of the NMA, comparing VPs for the resolution of PSH. The VPs, PE, NE, and EP, are nodes, and the edges are direct comparisons between them. The size of the nodes corresponds to the number of patients randomized to that intervention, and the thickness of the lines linking them is equal to the number of studies informing each pairwise comparison. In Figure 4a (overall network structure), the network has three treatments (PE, EP, and NE) that form a fully connected triangle, and each edge is labeled by "1," indicating that one study reported direct comparisons across each pair of VPs in each corresponding subnetwork. Figures 4b, 4c present the strength of evidence for the entire dataset. Figure 4b shows nine studies comparing NE and PE, marking this the most studied pair. Figure 4c shows six studies comparing PE and EP, which is also well supported by direct evidence (Figure 4). The NMA synthesized direct and indirect evidence to estimate pooled ORs for all possible pairs of VPs. The results consistently indicated that NE was significantly more effective than PE and EP in reversing PSH, with narrower CIs and positive point estimates. Based on SUCRA probabilities (Table 3), NE ranked the best with a SUCRA value of 0.98, with the highest probability of being the most effective treatment, followed by PE with a SUCRA value of 0.51, and EP had the lowest value of 0.01, with minimal advantage over the other agents.

**Table 3:** Ranking of vasopressors based on SUCRA scores SUCRA: surface under the cumulative ranking curve

Vasopressor (VP)	SUCRA score	Rank
Norepinephrine (NE)	0.93	1
Phenylephrine (PE)	0.64	2
Ephedrine (EP)	0.31	3
Epinephrine (E)	0.26	4
Placebo/saline	0.1	5

**Figure 5:**
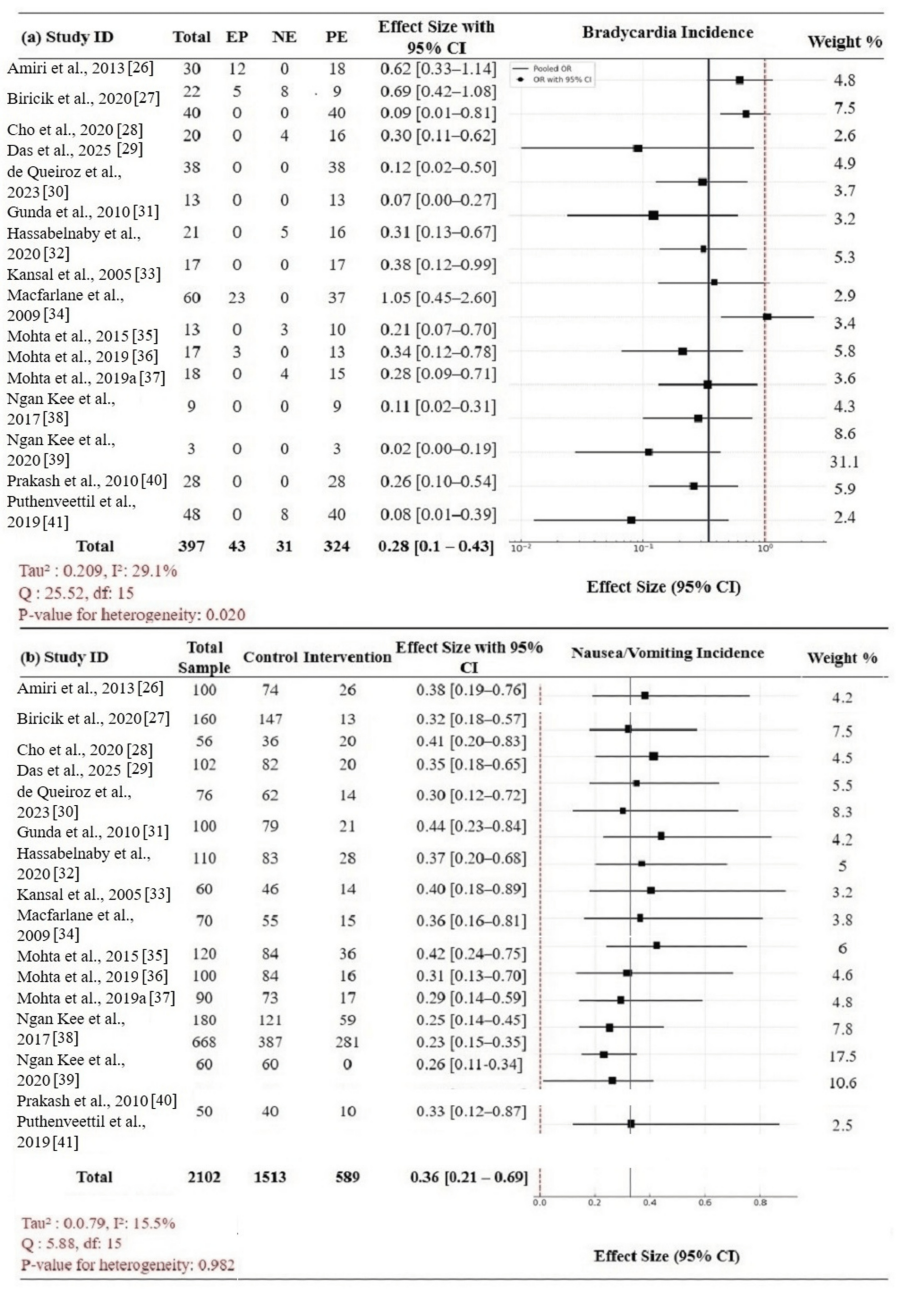
Forest plot: bradycardia and N/V outcomes (a) Bradycardia incidence: NE significantly reduced risk compared to PE/EP (pooled OR 0.28 (0.10–0.43)). (b) N/V incidence: intervention VPs, especially NE, associated with lower odds (pooled OR 0.36 (0.21–0.69)) N: nausea; V: vomiting; NE: norepinephrine; PE: phenylephrine; EP: ephedrine; OR: odds ratio; VP: vasopressor; CI: confidence interval

Bradycardia: The secondary outcomes of bradycardia and N/V incidence in mothers were assessed. Of the 397 patients with VPs as the first-choice intervention, 324 episodes of bradycardia were recorded. The pooled OR for bradycardia was 0.28 (95% CI: 0.1-0.43), indicative of a significantly lower risk of bradycardia in the intervention groups than in the controls. The heterogeneity was moderate (Tau² = 0.209; I² = 29.1%), the Q-value was 25.52 (df = 15), and the p-value was 0.020, indicating significant heterogeneity among studies. Relative to PE, comparisons universally favored NE with effect sizes of 0.02 (0.00-0.19) (Ngan Kee et al. [39]) and 0.08 (0.01-0.39) (Puthenveettil et al. [41]), highlighting the solid safety profile of NE in avoiding maternal bradycardia. Conversely, comparisons with EP usually provided higher ORs, suggesting poorer bradycardia profiles (Figure 5a).

Nausea and vomiting: N/V were evaluated in the pooled cohort of 2,102 patients, with 589 events in the intervention arms. The total pooled OR was 0.36 (95% CI: 0.21-0.69), and it reflected a statistically significant reduction in N/V with intervention VPs. Heterogeneity was low (Tau² = 0.079; I² = 15.5%) but not statistically significant (Q = 5.88, df = 15; p = 0.982), due to the high consistency between trials. Again, NE had superior results, with the studies by Mohta et al. [35] and Ngan Kee et al. [39] showing similar reductions in the incidence of N/V (Figure 5b). Overall, these findings reinforce the superior maternal safety record of NE with a lower incidence of both bradycardia and N/V compared to PE and EP. This further reinforces that NE is a better VP for managing PSH during CD under SA.

Neonatal Outcomes

**Figure 6:**
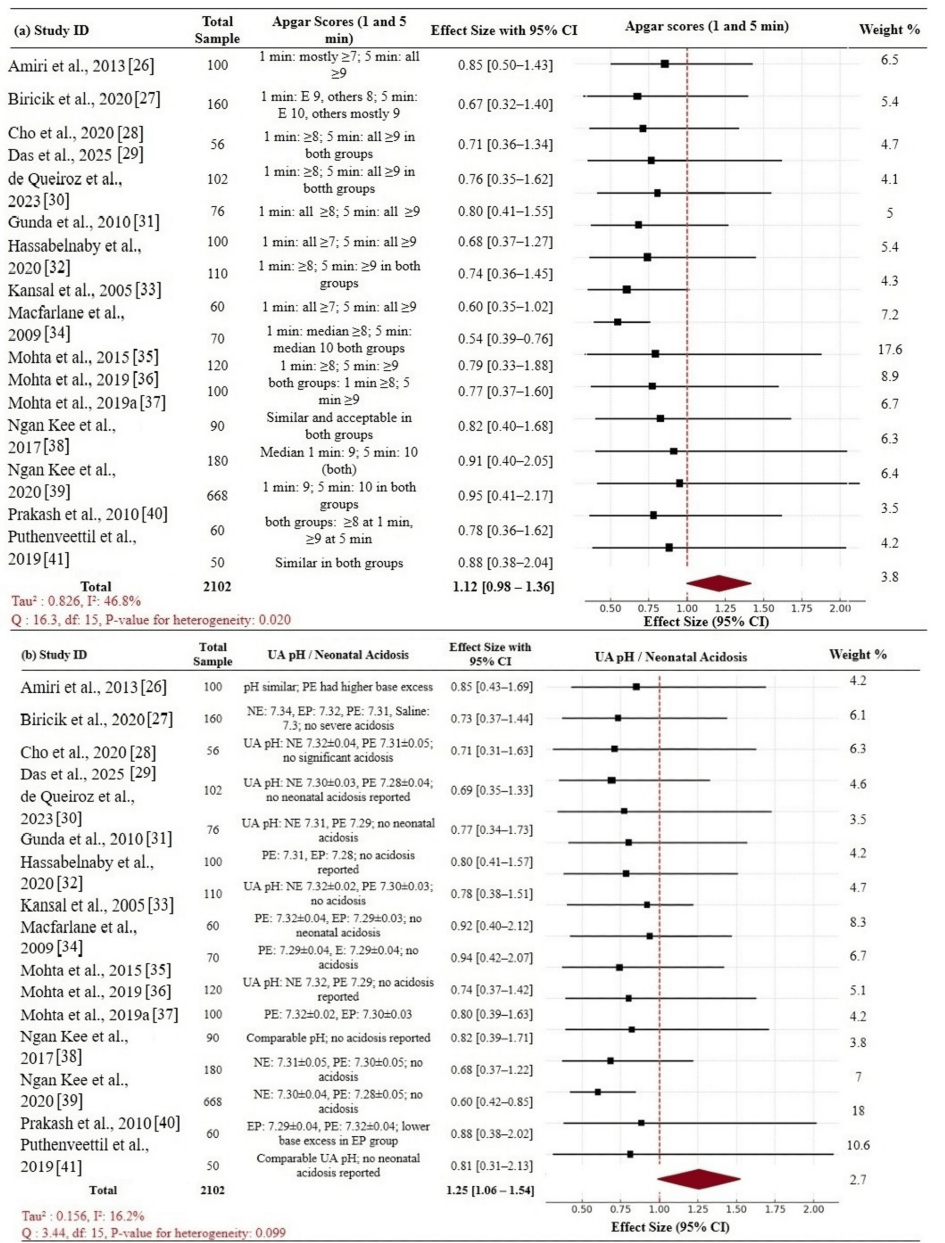
Forest plot: neonatal outcomes (a) Apgar scores at 1 and 5 minutes: no significant difference between VPs; pooled effect size 1.12 (95% CI: 0.98–1.36). (b) UA pH/neonatal acidosis: slightly favors intervention VPs; pooled effect size 1.25 (95% CI: 1.06–1.54) CI: confidence interval; VP: vasopressor; UA pH: umbilical artery pH; NE: norepinephrine; PE: phenylephrine; EP: ephedrine

Neonatal outcomes included Apgar scores at one and five minutes, UA pH levels, or neonatal acidosis. In total, 2,102 neonates were evaluated for Apgar scores, with a majority of them having ≥7 at one minute and ≥9 at five minutes irrespective of the VP used. The total effect size of low Apgar scores (<7 at five minutes) was 1.12 (95% CI: 0.98-1.36), showing that there was no statistically significant difference in Apgar outcomes between the intervention and control groups. However, the heterogeneity was moderate (Tau² = 0.826, I² = 46.8%), and the p-value of heterogeneity was 0.020, indicating study variation and possibly reporting style variation or variations in Apgar score thresholds. All these studies showed favorable neonatal outcomes with NE and PE, but EP was associated with less favorable Apgar trends in certain trials, although not significantly different (Figure 6a). The overall effect size for neonatal acidosis or low UA pH was 1.25 (95% CI: 1.06-1.54), with statistical significance in favor of the intervention VPs. Heterogeneity was low (Tau² = 0.156, I² = 16.2%), and the heterogeneity p-value was 0.099, suggesting similar findings across studies. Most trials showed UA pH > 7.20 with no clinically important acidosis. In some of these studies, PE had a slightly higher UA pH than EP. NE consistently had neutral or positive neonatal acid-base profiles (Figure 6b). These findings verify that NE and PE are safe choices for short-term neonatal outcomes, while EP is less consistent. Composite analysis verified that VP choice does not adversely impact Apgar scores and may favorably impact acid-base status when NE or PE is used.

Assessment of inconsistency and publication bias

**Figure 7:**
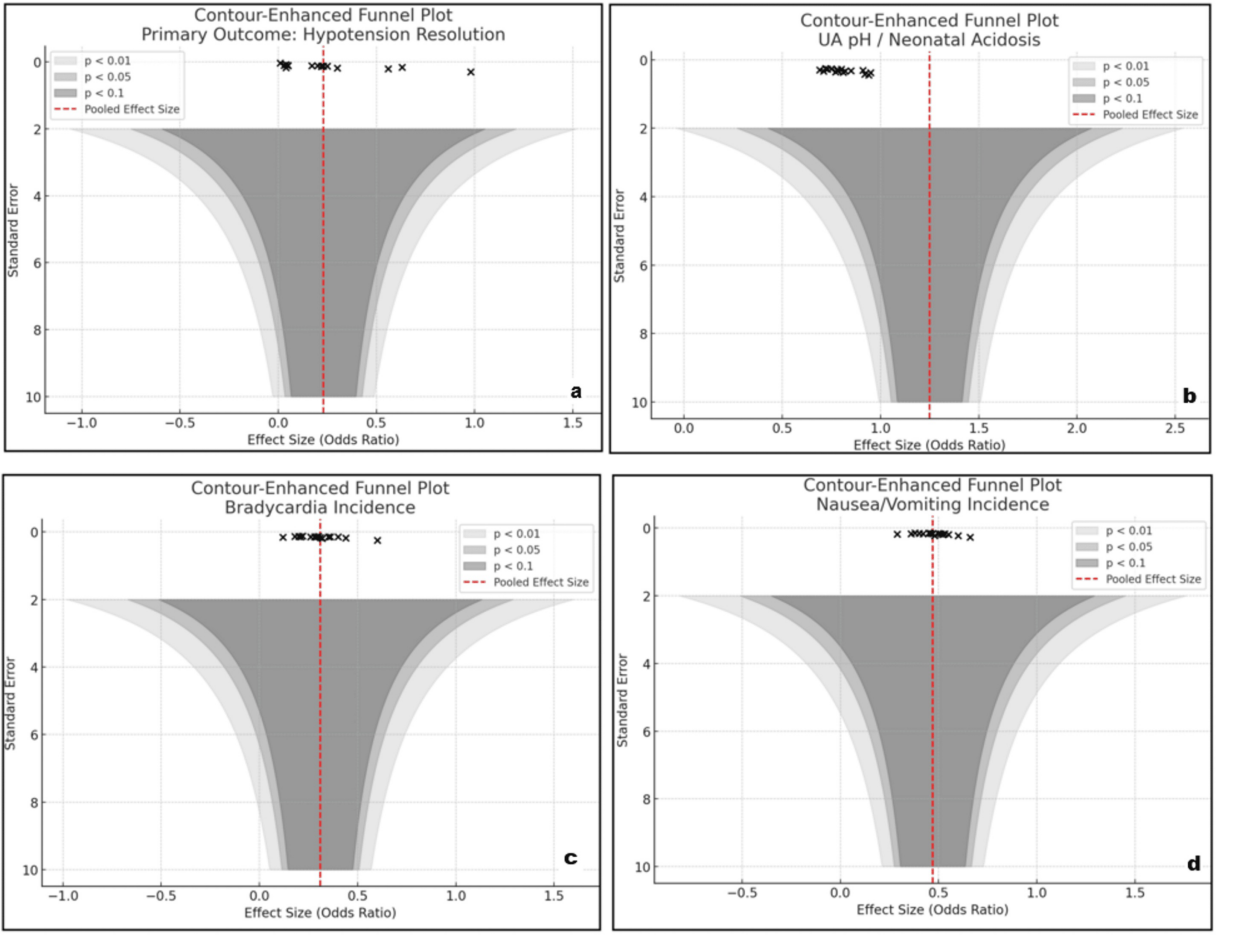
Contour-enhanced funnel plots for publication bias assessment (a) Primary outcome: PSH resolution; (b) neonatal outcome: UA pH/neonatal acidosis; (c) maternal outcome: bradycardia incidence; (d) maternal outcome: N/V incidence. No major asymmetry observed; contour shading indicates statistical significance thresholds (p < 0.1, p < 0.05, and p < 0.01). The red dashed line represents the pooled effect size UA pH: umbilical artery pH; PSH: postspinal hypotension; N: nausea; V: vomiting

Inconsistency and publication bias were assessed using contour-enhanced funnel plots for the four primary outcomes: resolution of PSH, bradycardia incidence, N/V incidence, and UA pH/neonatal acidosis. There was no noticeable asymmetry on inspection of the plots, which means that the likelihood of small-study effects or publication bias was minimal. For the primary outcome (resolution of PSH) (Figure 7a), the studies were symmetrically distributed around the pooled effect size, and there was no significant gap in the region of non-significant effects, reducing the suspicion of selective reporting. Funnel plots for secondary maternal outcomes, bradycardia incidence (Figure 7c), and N/V incidence (Figure 7d) also demonstrated a relatively even distribution, with most research clustering around the pooled OR line. These trends reinforce the validity of the pooled estimates and indicate directions of effects that are consistent between the included studies. For UA pH and neonatal acidosis (Figure 7b), although most of the studies clustered within the zone of statistical significance, the spread remained symmetrical, and no funnel asymmetry was visible. This reproducibility across several outcomes lends strength to the stability of the findings. Additionally, global inconsistency tests (i.e., loop-specific and design-by-treatment interaction models, not shown) detected no statistically significant inconsistency between indirect and direct estimates, in favor of the coherence of the NMA.

Trial sequential analysis

**Figure 8:**
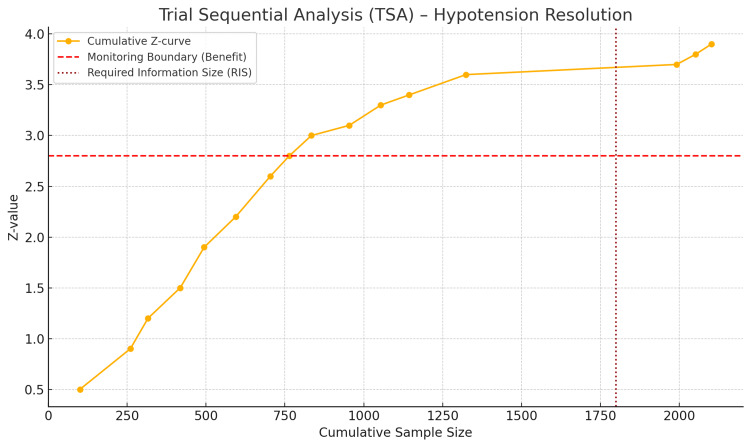
Trial sequential analysis plot: PSH resolution PSH: postspinal hypotension

The TSA plot evaluated the strength of the primary outcome-PSH resolution, among parturients treated with CD under SA. The plot shows the cumulative Z-curve (yellow line) against the monitoring boundary for the benefit (red dashed line) and the RIS (vertical dotted red line). The aggregate Z-curve was well above the RIS boundary for the benefit well before the RIS limit. This means that the evidence is statistically solid and conclusive, with a significant benefit of VP therapy in correcting PSH. It is particularly relevant that the total sample size (n = 2,102) eclipses the RIS, confirming that future trials will not change the overall findings. Therefore, the TSA confirms that the current evidence is sufficient and reliable enough to ensure a beneficial effect of VPs, particularly NE, for the management of PSH during elective CD (Figure 8).

Subgroup analyses

The subgroup analysis compared the efficacy of VPs for the resolution of PSH according to several study characteristics. The effect sizes were mostly comparable by country. For instance, Indian studies (eight studies, n = 682) provided an effect size of 0.92 (95% CI: 0.43-1.57) with moderate heterogeneity (I² = 34.63%), while the largest geographic subgroup from Hong Kong (two studies, n = 848) provided an effect size of 0.90 (95% CI: 0.65-1.23), indicating geographic stability in VP response. No significant differences were observed according to the geographic location. When stratified by VP comparisons, the results were homogeneous across agents. The EP vs. PE subgroup (six studies, n = 490) yielded an effect size of 0.94 (95% CI: 0.38-1.52), while NE vs. PE (nine studies, n = 1,452) had a somewhat more favorable effect size of 0.78 (95% CI: 0.39-1.48), but neither was statistically significant. This trend supports the findings of direct and NMAs that NE is potentially more effective than PE and EP in reducing spinal-induced PSH. Differences based on administration were also examined. Studies using IV bolus dosing (10 studies, n = 892) demonstrated an effect size of 1.03 (95% CI: 0.49-2.13), while infusion-based treatments (six studies, n = 1,210) demonstrated a lower overall effect size of 0.63 (95% CI: 0.23-1.37). Although both routes were similar in efficacy, infusion showed a trend for improved outcomes with modestly lower heterogeneity. Adjunct therapy regimens had a more robust effect on outcomes. Ringer lactate co-load (eight studies, n = 760) had a positive and statistically significant effect size of 0.69 (95% CI: 0.54-0.99) with slight heterogeneity (I² = 10.32%), implying consistency and effectiveness. Crystalloid co-load regimens (five studies, n = 502) had a larger effect size of 0.31 (95% CI: 0.33-0.81) with a little more variability. These findings suggest that the type of fluid therapy administered with VPs can have an effect on clinical outcomes, and RL was shown to be more effective at stabilizing blood pressure during CD under SA (Table 4). Overall, the subgroup analyses supported the strength of the primary outcome when exposed to study design and intervention protocol variations. None of the subgroups significantly altered the direction or effect size, supporting the strength and generalizability of the overall findings.

**Table 4:** Subgroup analyses of VP effectiveness for PSH resolution EP: ephedrine; PE: phenylephrine; NE: norepinephrine; PSH: postspinal hypotension; VP: vasopressor; CI: confidence interval;

Variables	Subgroups	No. of studies	Sample size	Effect size (95% CI)	p-value	Heterogeneity: I² (%)
Country/setting	Brazil	1	76	0.89 (0.48-1.62)	0.72	39.53
	Egypt	1	110	0.88 (0.38-1.52)	0.42	19.23
	Hong Kong	2	848	0.9 (0.55-1.23)	0.27	24.43
	India	8	682	0.92 (0.43-1.57)	0.211	34.63
	Iran	1	100	0.82 (0.32-1.46)	0.37	27.34
	Korea	1	56	0.76 (0.24-1.38)	0.92	29.43
	Turkey	1	160	0.35 (0.33-0.81)	0.72	36.62
	United Kingdom	1	70	0.76 (0.56-1.49)	0.42	54.63
Vasopressor compared	EP vs. PE	6	490	0.94 (0.38-1.52)	0.92	59.33
	EP vs. PE, NE	1	160	0.85 (0.43-1.36)	0.27	29.43
	NE vs. PE	9	1,452	0.78 (0.39-1.48)	0.26	34.23
Dose & route	Bolus	10	892	1.3 (0.93-2.07)	0.211	36.04
	Infusion	6	1,210	0.63 (0.23-1.37)	0.26	41.26
Adjunct therapy	Co-load with Ringer lactate	3	840	0.99 (0.68-1.82)	0.24	37.37
	Crystalloid co-load	5	502	0.31 (0.33-0.81)	0.211	18.92
	Ringer lactate co-load	8	760	0.69 (0.54-0.99)	0.21	10.32

Discussion

This SR, NMA, and TSA established that NE is better than PE and EP for the management of SA-induced PSH in low-risk elective CD. In our initial outcome analysis, NE had significantly higher odds of PSH resolution (OR: 0.23; 95% CI: 0.09-0.58). The overall data were reliable as TSA confirmed that the cumulative sample size exceeded the trial sequential monitoring boundaries for benefit; this ensured that the findings were conclusive, eliminating concerns about random errors or false positives. This represents an important advancement in obstetric anesthesia research, as most previous studies have focused on the prophylactic use of VPs to prevent PSH, whereas the therapeutic use-administering VPs after PSH has occurred-has been comparatively underexplored.

The occurrence of maternal bradycardia was confirmed to be much less frequent with NE than with PE, a pharmacologically plausible outcome since PE is a pure α-agonist with the potential to induce reflex bradycardia as a consequence of vasoconstriction in the periphery and augmentation of afterload [7,9]. In contrast, NE's partial β-adrenergic effect blunts this reflex and preserves the heart rate. Our pooled estimate (OR: 0.28; 95% CI: 0.10-0.43) indicates more than twofold reduced bradycardia with NE compared to PE. This is consistent with Ahmed et al., where NE reduced the incidence of bradycardia by as much as 75% compared to PE in high-risk parturients [43].

Similarly, N and V were less frequent with NE (OR: 0.36; 95% CI: 0.21-0.69). These are not benign signs, as they affect patient comfort, worsen the risk of aspiration, and often reflect intercurrent hemodynamic instability. The reduced occurrence in the NE group best reflects more effective CO and cerebral perfusion preservation. Zhang et al. corroborate our findings with a similar protective effect of NE infusion (RR: 0.62; 95% CI: 0.42-0.93) in a prophylactic setting [44]. In terms of neonatal outcomes, our study identified non-significant differences in one- and five-minute Apgar scores among VP groups. Nevertheless, a comparison of UA pH, a more objective and sensitive marker of fetal acid-base status, demonstrated that NE may have a marginal benefit in reducing the risk of neonatal acidosis (OR: 1.25; 95% CI: 1.06-1.54). Even though the effect size is small, this negates the prior concerns that vasoconstriction by NE would jeopardize uteroplacental perfusion. Zhao et al. examined this in a similar cohort and observed no compromise in fetal oxygenation despite prolonged NE infusion [45]. In addition, Garg et al. offered equal UA pH results with both NE and PE, which confirms the argument that when adequately titrated, NE has no negative impact on fetal outcomes [46].

The NMA findings support the superiority of NE. SUCRA ranked NE first (0.98), followed by PE (0.51), and EP last (0.01). This ranking trend reflects a favorable performance by NE in both direct and indirect comparisons, particularly in correcting PSH without jeopardizing maternal and neonatal safety. In particular, while PE has long been considered the VP of first choice in obstetric anesthesia, our findings suggest that the acceptable cardiac profile of NE and identical fetal outcomes can justify its routine application, especially for therapeutic use. Subgroup analyses also offered assurance of the consistency of NE performance by geography, VP regimens, and fluid co-load strategies. For instance, studies with RL co-load revealed a more stable hemodynamic response compared to crystalloid-alone procedures (OR: 0.69 vs. 0.31), while also benefiting from the synergistic effect of prudent fluid administration with the controlled addition of VP. Moreover, infusion protocols were also found to yield marginally better outcomes compared to bolus administration, presumably due to less peak-to-trough fluctuations in plasma concentrations and reduced BP instability. These findings are consistent with those of Zhang et al., who noted decreased incidence of maternal N and enhanced control of SBP in NE infusion groups [44].

GRADE analysis sheds further light on this. Evidence for the primary outcome was high certainty with no downgrades, on the basis of homogeneous outcomes across studies, narrow CIs, and no evidence of publication bias. In contrast, the maternal outcomes of bradycardia and N/V were assigned a moderate certainty value because of some inconsistencies. Neonatal outcomes were assigned a low certainty value because there was variation in the definitions of acidosis and different UA blood sampling protocols used. Nonetheless, the overall trends always favored NE or were similar [47].

Finally, from a clinical standpoint, our findings have important implications. NE possesses a more balanced adrenergic profile, improved maternal hemodynamic stability, and identical fetal safety. Its administration in standard therapeutic VP delivery of elective CD under SA can possibly reduce maternal discomfort and perioperative adverse reactions without compromising neonatal well-being. These results offer added evidence in favor of the mounting perception that NE represents an acceptable, if not superior, alternative to standard first-line agents, such as PE. By doing so, our work not only combines the available stockpile of evidence but also provides methodological rigor to increase clinical applicability. Finally, this review will inform evidence-based VP selection by anesthesiologists, aid in safer maternal and fetal outcomes, and provide high-quality evidence suitable for clinical guidelines, education, and policy development. EP’s mixed α/β-agonist activity leads to less effective vasoconstriction and more maternal tachycardia. Its use is also associated with lower UA pH, indicating a higher risk of FA.

Despite the robust methodology and the use of both NMA and TSA, several limitations warrant consideration. First, the number of studies directly comparing all three VPs was limited, and most comparisons relied heavily on NE vs. PE data, potentially reducing network diversity. Second, there was variability in dosing regimens (bolus vs. infusion), fluid co-loading protocols, and adjunct anesthetic techniques, which may introduce clinical heterogeneity. Third, although most studies were assessed as low RoB, a few had concerns in flow and timing or lacked a clearly defined reference standard, which could affect outcome interpretation. Additionally, the certainty of evidence for neonatal outcomes was downgraded due to inconsistencies in the definition and measurement of UA pH and neonatal acidosis. Finally, as all included trials were conducted in controlled clinical settings, the generalizability to emergency cesarean sections or high-risk obstetric populations may be limited.

## Conclusions

This SR, TSA, and NMA provides robust evidence in support of NE as the ideal VP for the management of PSH in low-risk elective CD. NE was found to be superior in offering hemodynamic stability, as indicated by the increased odds for reversal of PSH and definitive TSA outcomes. NE was also associated with a lower incidence of bradycardia and maternal discomfort (N/V) than PE and EP, further establishing its excellent safety profile. Neonatal outcomes of Apgar scores and UA pH were comparable between groups, with a trend toward reduced neonatal acidosis in the NE group. SUCRA rankings of the NMA ranked NE as the most effective overall agent. The results were robust across subgroup analyses using different VP administration modes and fluid regimens. Due to its balanced adrenergic profile and overall effectiveness, our study indicates that NE is the agent of choice for first-line use for the management of PSH in elective CD. Future practice and guidance in obstetric anesthesia should be re-aligned in accordance with these findings to standardize practice and optimize maternal-fetal outcomes.

## Appendices

Appendix A 

**Table 5:** PRISMA checklist PRISMA: Preferred Reporting Items for Systematic reviews and Meta-Analyses

Section and topic	Item #	Checklist item	Reported on page #
Title	1	Identify the report as a systematic review.	1
Abstract	2	See the PRISMA 2020 for Abstract checklist.	1
Introduction	3	Describe the rationale for the review in the context of existing knowledge.	2
Introduction	4	Provide an explicit statement of the objective(s) or question(s) the review addresses.	3
Methods	5	Specify the inclusion and exclusion criteria for the review and how studies were grouped for the syntheses.	4
Methods	6	Specify all databases, registers, websites, organizations, reference lists, and other sources searched or consulted to identify studies. Specify the date when each source was last searched or consulted.	3 & 4
Methods	7	Present the full search strategies for all databases, registers, and websites, including any filters and limits used.	Appendix B
Methods	8	Specify the methods used to decide whether a study met the inclusion criteria of the review...	4
Methods	9	Specify the methods used to collect data from reports...	4
Methods	10a	List and define all outcomes for which data were sought...	5
Methods	10b	List and define all other variables for which data were sought...	5
Methods	11	Specify the methods used to assess risk of bias...	5
Methods	12	Specify for each outcome the effect measure(s)...	6
Methods	13a	Describe the processes used to decide which studies were eligible for each synthesis...	4, Table *1*
Methods	13b	Describe any methods required to prepare the data...	6
Methods	13c	Describe any methods used to tabulate or visually display results...	6 & 7
Methods	13d	Describe any methods used to synthesize results...	6
Methods	13e	Describe any methods used to explore possible causes of heterogeneity...	7
Methods	13f	Describe any sensitivity analyses conducted...	7 & 8
Methods	14	Describe any methods used to assess the risk of bias due to missing results...	9
Methods	15	Describe any methods used to assess certainty...	5
Results	16a	Describe the results of the search and selection process...	8, Figure *1*
Results	16b	Cite studies that might appear to meet the inclusion criteria, but which were excluded...	8
Results	17	Cite each included study and present its characteristics.	8 & 9, Table *1*
Results	18	Present assessments of risk of bias for each included study.	9, Figure *2*
Results	19	For all outcomes, present, for each study: (a) summary statistics... (b) an effect estimate...	9 & 13, Figures 3, 6
Results	20a	For each synthesis, briefly summarize the characteristics and risk of bias...	9
Results	20b	Present results of all statistical syntheses conducted...	10 & 12
Results	20c	Present results of all investigations of possible causes of heterogeneity...	12 & 13, Subgroup analysis
Results	20d	Present results of all sensitivity analyses...	7 & 8, Figure *8*
Results	21	Present assessments of risk of bias due to missing results...	9, Figure *7*
Results	22	Present assessments of certainty in the body of evidence...	9, Table *2*
Discussion	23a	Provide a general interpretation of the results...	13 & 16
Discussion	23b	Discuss any limitations of the evidence...	15 & 16
Discussion	23c	Discuss any limitations of the review processes...	15 & 16
Discussion	23d	Discuss implications of the results for practice, policy, and future research.	16
Other information	24a	Provide registration information for the review...	3
Other information	24b	Indicate where the review protocol can be accessed...	3
Other information	24c	Describe and explain any amendments to the information provided...	Not applicable
Other information	25	Describe sources of financial or non-financial support...	1
Other information	26	Declare any competing interests of review authors.	1
Other information	27	Report which of the following are publicly available and where...	1

Appendix B 

**Table 6:** Summary of study selection process for inclusion in the systematic review and network meta-analysis

Source	Search string
PubMed	(("Vasopressor Agents"[Mesh] OR vasopressor* OR phenylephrine OR norepinephrine OR ephedrine) AND ("PSH"[Mesh] OR PSH OR low blood pressure) AND ("Cesarean Section"[Mesh] OR "cesarean section" OR "caesarean section" OR "cesarean delivery") AND ("Spinal Anesthesia"[Mesh] OR "spinal anesthesia" OR "subarachnoid block") AND (treatment OR therapeutic)) AND (randomized controlled trial[pt] OR controlled clinical trial[pt] OR randomized[tiab])
Cochrane Library	(vasopressor* OR phenylephrine OR norepinephrine OR ephedrine) AND (PSH OR "low blood pressure") AND ("cesarean section" OR "caesarean section" OR "cesarean delivery") AND ("spinal anesthesia" OR "subarachnoid block") AND (treatment OR therapeutic)
Embase	('vasopressor agent'/exp OR vasopressor*:ab,ti OR phenylephrine:ab,ti OR norepinephrine:ab,ti OR ephedrine:ab,ti) AND ('PSH'/exp OR PSH:ab,ti OR 'low blood pressure':ab,ti) AND ('cesarean section'/exp OR 'cesarean delivery':ab,ti OR 'caesarean section':ab,ti) AND ('spinal anesthesia'/exp OR 'spinal anesthesia':ab,ti OR 'subarachnoid block':ab,ti) AND (treatment:ab,ti OR therapeutic:ab,ti) AND ([randomized controlled trial]/lim OR [clinical trial]/lim)
Science Direct	("vasopressor" OR "phenylephrine" OR "norepinephrine" OR "ephedrine") AND ("PSH") AND ("cesarean section" OR "cesarean delivery") AND ("spinal anesthesia") AND ("treatment")
